# Tri­phenyl­phospho­nium tri­chlorido­(tri­phenyl­phosphane-κ*P*)cobaltate(II) benzene disolvate

**DOI:** 10.1107/S2414314624011210

**Published:** 2024-11-22

**Authors:** Lubabalo Ndima, Eric Cyriel Hosten, Richard Betz

**Affiliations:** a Nelson Mandela University, Summerstrand Campus, Department of Chemistry, University Way, Summerstrand, PO Box 77000, Port Elizabeth, 6031, South Africa; Vienna University of Technology, Austria

**Keywords:** crystal structure, salt, cobaltate(II), hydrogen bonding

## Abstract

The solvated title compound, (C_18_H_16_P)[CoCl_3_(C_18_H_15_P)]·2C_6_H_6_, is the tri­phenyl­phospho­nium salt of an anionic Co^II^ chlorido coordination compound; the asymmetric unit features an ion-pair and two benzene solvent molecules. One of the solvent mol­ecules shows rotational disorder. C—H⋯Cl and P—H⋯Cl contacts connect the individual constituents into infinite chains extending parallel to [010].

## Abstract 2

The central atom of the complex anion is coordinated by one tri­phenyl­phosphane and three chlorido ligands. The negative charge is balanced by a tri­phenyl­phospho­nium cation. Two benzene solvent mol­ecules are also present in the asymmetric unit.

## Graphical abstract

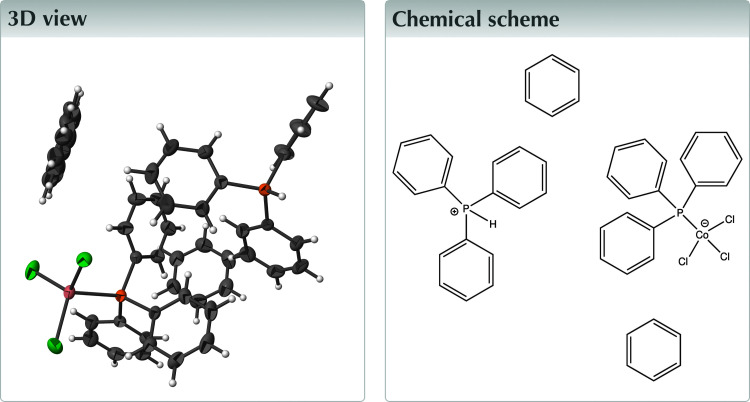

## Structure description

Coordination compounds of transition metals play a crucial role in a multitude of industrial and laboratory synthesis protocols. The nature of the metal, the ligands and electronic configuration can be tweaked systematically to optimize reactivity (Gade, 1998 ▸). In our ongoing inter­est into coordination compounds featuring halogenido ligands of main group metals such as anti­mony (Averdunk *et al.*, 2021 ▸) as well as transition metals such as rhenium (Yumata *et al.*, 2011 ▸; Schoultz *et al.*, 2016 ▸; Gerber *et al.*, 2011 ▸), iron (Schlamp *et al.*, 2012 ▸), zinc (Hosten *et al.*, 2015*a* ▸), copper (Hosten & Betz, 2016 ▸; Moosun *et al.*, 2015 ▸) and cobalt (Hosten *et al.*, 2015*b* ▸), we sought to expand our knowledge into the field of anionic cobalt coordination compounds featuring phospho­nium counter-ions, especially protonated tri­phenyl­phosphane. While not that common, some structural information about the latter class of ionic compounds is apparent in the literature, predominantly for halogenido coordination compounds of several heavier *d*-block elements such as molybdenum (Junk & Atwood, 1999 ▸), tungsten (Bhuiyan *et al.*, 2015 ▸) and osmium (Robinson *et al.*, 1988 ▸) as well as selected lanthanides such as praseodymium (Majeste *et al.*, 1977 ▸) and main-group-based coordination compounds involving, among others, phospho­rus (Dyke *et al.*, 2020 ▸) and boron (Burke *et al.*, 2020 ▸).

In the crystal structure of the title compound, a tetra­coordinate Co^II^ atom is present whose ligand sphere is comprised of one tri­phenyl­phosphane as well as three chlorido ligands. The negative charge of the cobaltate is balanced by one tri­phenyl­phospho­nium cation. Furthermore, the asymmetric unit contains two benzene solvent mol­ecules, one of which shows rotational disorder over two positions.

**Figure 1:**
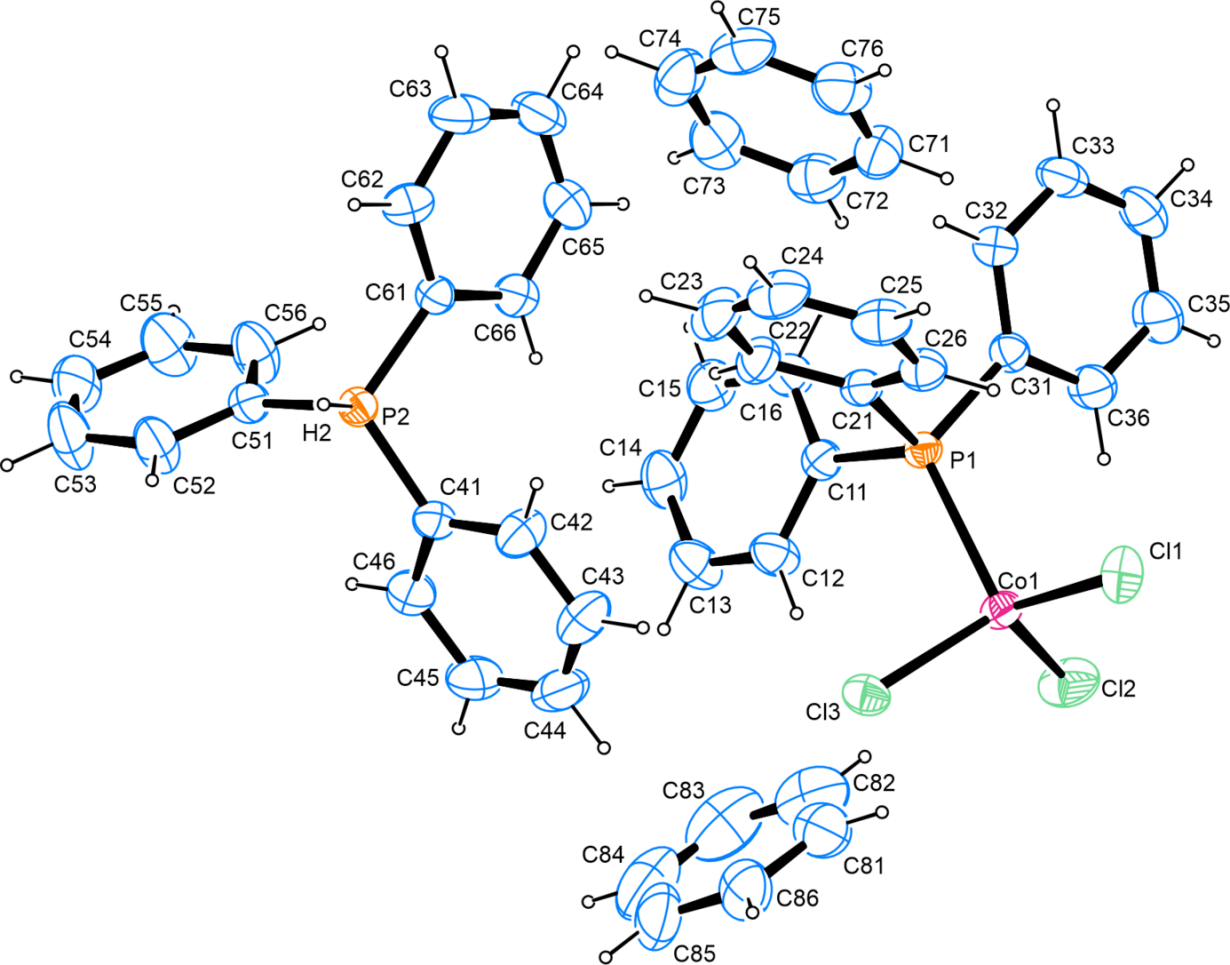
The structures of the mol­ecular entities in the title compound, showing atom labels and anisotropic displacement ellipsoids drawn at the 50% probability level. For clarity, only the major component (C81–C86) of the disordered solvent mol­ecule is depicted.

The Co—Cl bond lengths cover a range of 2.2313 (5)–2.2671 (4) Å, and the Co—P bond length is 2.3893 (4) Å. Both findings are in good agreement with comparable cobalt coord­ination compounds whose metrical parameters have been determined on grounds of diffraction studies and deposited with the Cambridge Structural Database (Groom *et al.*, 2016 ▸). Inter­atomic angles over the central metal atom span 101.167 (15)–115.758 (19)°, which is indicative of a distorted tetra­hedral coordination sphere. The least-squares planes as defined by the respective carbon atoms of the aromatic systems of the cobalt-bound phosphane inter­sect at angles of 77.22 (8), 79.87 (8) and 80.08 (8)° while the corresponding angles in the protonated counter-ion present as 77.06 (9), 88.97 (10) and 89.52 (10)°, respectively (Fig. 1 ▸).

**Figure 2:**
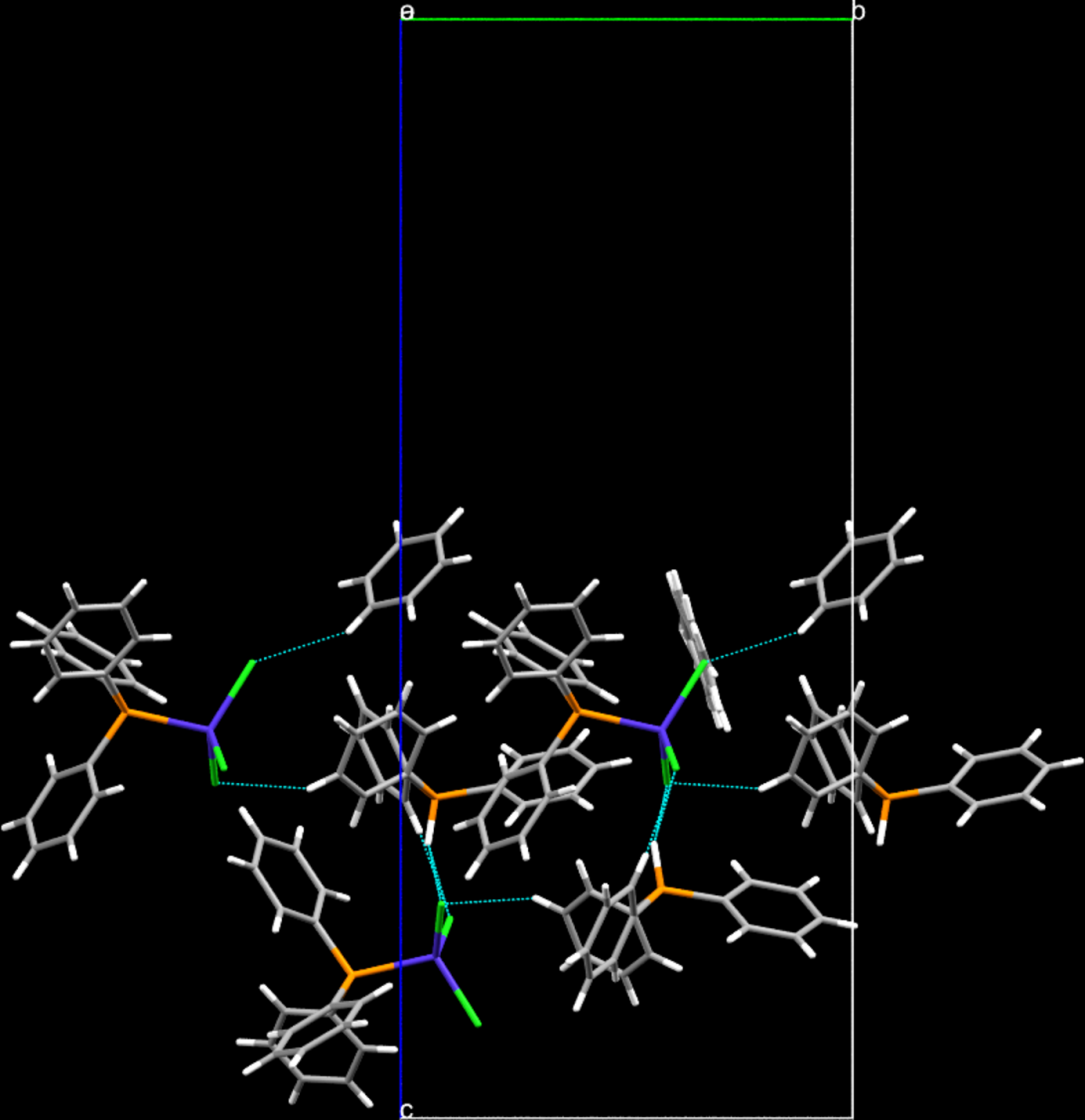
Inter­molecular contacts (shown as dashed lines) in the crystal structure of the title compound, in a view along [100].

In the crystal, C—H⋯Cl and P—H⋯Cl contacts, whose ranges fall by more than 0.1 Å below the sum of the van der Waals radii of the atoms participating in them, are apparent (Table 1 ▸). While the C—H⋯Cl contacts are supported by one hydrogen atom in the *ortho* (H52) as well as the *meta* (H63) positions on two different benzene rings of the cation, both solvent mol­ecules establish one C—H⋯Cl contact each. All chlorido ligands act as acceptors, one of them as a threefold acceptor (Table 1 ▸). The phospho­rus-bonded hydrogen atom (H2) acts as bifurcated donor towards two chlorido acceptors. In terms of graph-set analysis (Etter *et al.*, 1990 ▸; Bernstein *et al.*, 1995 ▸), the descriptor for these contacts is *DDDDDD* on the unary level. π-Stacking is not a prominent stabilizing feature in the crystal structure of the title compound with the shortest inter­centroid distance between two aromatic systems being 4.3603 (11) Å, which involves one phenyl group each on the protonated as well as on the metal-bonded tri­phenyl­phosphane moieties. In total, the individual mol­ecular entities are connected into infinite chains extending parallel to [010] by these latter contacts (Fig. 2 ▸). Furthermore, a number of C—H⋯π inter­actions (Table 1 ▸) are apparent in the crystal structure that are supported by one hydrogen atom each on the solvent mol­ecules as well as on one of the aromatic hydrogen atoms in the *ortho* position (H62) to the protonated phospho­rus atom as donors while the aromatic system of the disordered solvent mol­ecule and two of the aromatic systems of the coordinating tri­phenyl­phosphane mol­ecule serve as acceptors.

## Synthesis and crystallization

The title compound was obtained by reacting bis­(tri­phenyl­phosphane)cobalt(II) chloride and the hydrido­spiro­phosphor­ane derived from α-hy­droxy-cyclo­penta­necarb­oxy­lic acid in the presence of *n*-butyl­lithium in THF/benzene. Crystals suitable for the diffraction study were obtained upon concentrating the reaction mixture and subsequent storage at room temperature.

## Refinement

Crystallographic data and structure refinement details are summarized in Table 2 ▸. The H atom of the phospho­nium cation was located from a difference-Fourier map and refined freely. The modelling of the disordered benzene mol­ecule was conducted applying RIGU instructions; the refined split ratio is 0.580 (11):0.420 (11) for atoms (C81–C86):(C91–C96). Reflections 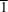
02 and 100 were obstructed by the beam stop and were omitted from refinement.

## Supplementary Material

Crystal structure: contains datablock(s) I. DOI: 10.1107/S2414314624011210/wm4224sup1.cif

Structure factors: contains datablock(s) I. DOI: 10.1107/S2414314624011210/wm4224Isup2.hkl

CCDC reference: 2403788

Additional supporting information:  crystallographic information; 3D view; checkCIF report

## Figures and Tables

**Table 1:** Hydrogen-bond geometry (Å, °) *Cg*1–*Cg*4 are the centroids of the (C21–C26), (C81–C86), (C91–C96) and (C11–C16) rings, respectively.

*D*—H⋯*A*	*D*—H	H⋯*A*	*D*⋯*A*	*D*—H⋯*A*
P2—H2⋯Cl1^i^	1.271 (18)	2.701 (18)	3.7531 (6)	138.9 (11)
P2—H2⋯Cl3^i^	1.271 (18)	2.758 (18)	3.6380 (6)	124.9 (11)
C52—H52⋯Cl1^i^	0.95	2.80	3.6874 (19)	155
C63—H63⋯Cl3^ii^	0.95	2.76	3.6294 (19)	152
C75—H75⋯Cl2^ii^	0.95	2.85	3.694 (2)	149
C85a—H85a⋯Cl1^iii^	0.95	2.83	3.690 (3)	150
C62—H62⋯*Cg*1^i^	0.95	2.72	3.6042 (19)	154
C72—H72⋯*Cg*2^iv^	0.95	2.77	3.611 (3)	148
C72—H72⋯*Cg*3^iv^	0.95	2.78	3.607 (4)	146
C92—H92⋯*Cg*4^iv^	0.95	2.84	3.636 (6)	143

Symmetry codes: (i) 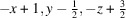; (ii) 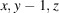; (iii) 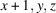; (iv) 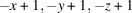.

**Table 2:** Experimental details

Crystal data	
Chemical formula	(C_18_H_16_P)[CoCl_3_(C_18_H_15_P)]·2C_6_H_6_
*M*_r_	847.04
Crystal system, space group	Monoclinic, *P*2_1_/*c*
Temperature (K)	200
*a*, *b*, *c* (Å)	11.1017 (5), 12.6011 (7), 30.7804 (17)
β (°)	96.1058 (18)
*V* (Å^3^)	4281.6 (4)
*Z*	4
Radiation type	Mo *K*α
μ (mm^−1^)	0.70
Crystal size (mm)	0.54 × 0.35 × 0.19

Data collection	
Diffractometer	Bruker D8 Quest CCD
Absorption correction	Multi-scan (*SADABS*; Krause *et al.*, 2015 ▸)
*T*_min_, *T*_max_	0.666, 0.746
No. of measured, independent and observed [*I* > 2σ(*I*)] reflections	152138, 10618, 9173
*R*_int_	0.032
(sin θ/λ)_max_ (Å^−1^)	0.667

Refinement	
*R*[*F*^2^ > 2σ(*F*^2^)], *wR*(*F*^2^), *S*	0.032, 0.081, 1.10
No. of reflections	10618
No. of parameters	523
No. of restraints	72
H-atom treatment	H atoms treated by a mixture of independent and constrained refinement
Δρ_max_, Δρ_min_ (e Å^−3^)	0.32, −0.39

Computer programs: *APEX2* and *SAINT* (Bruker, 2016 ▸), *SHELXS97* (Sheldrick, 2008 ▸), *ORTEP-3 for Windows* (Farrugia, 2012 ▸), *Mercury* (Macrae *et al.*, 2020 ▸), *SHELXL2019/3* (Sheldrick, 2015 ▸) and *PLATON* (Spek, 2020 ▸).

## full crystallographic data

### Triphenylphosphonium trichlorido(triphenylphosphane-κP)cobaltate(II) benzene disolvate Crystal data

**Table:**

(C_18_H_16_P)[CoCl_3_(C_18_H_15_P)]·2C_6_H_6_	*F*(000) = 1756
*M**_r_* = 847.04	*D*_x_ = 1.314 Mg m^−^^3^
Monoclinic, *P*2_1_/*c*	Mo *K*α radiation, λ = 0.71073 Å
*a* = 11.1017 (5) Å	Cell parameters from 9084 reflections
*b* = 12.6011 (7) Å	θ = 2.6–28.3°
*c* = 30.7804 (17) Å	µ = 0.70 mm^−^^1^
β = 96.1058 (18)°	*T* = 200 K
*V* = 4281.6 (4) Å^3^	Block, blue
*Z* = 4	0.54 × 0.35 × 0.19 mm

### Triphenylphosphonium trichlorido(triphenylphosphane-κP)cobaltate(II) benzene disolvate Data collection

**Table:**

Bruker D8 Quest CCD diffractometer	9173 reflections with *I* > 2σ(*I*)
φ and ω scans	*R*_int_ = 0.032
Absorption correction: multi-scan (SADABS; Krause *et al.*, 2015)	θ_max_ = 28.3°, θ_min_ = 2.1°
*T*_min_ = 0.666, *T*_max_ = 0.746	*h* = −14→13
152138 measured reflections	*k* = −16→16
10618 independent reflections	*l* = −41→41

### Triphenylphosphonium trichlorido(triphenylphosphane-κP)cobaltate(II) benzene disolvate Refinement

**Table:**

Refinement on *F*^2^	Secondary atom site location: difference Fourier map
Least-squares matrix: full	Hydrogen site location: mixed
*R*[*F*^2^ > 2σ(*F*^2^)] = 0.032	H atoms treated by a mixture of independent and constrained refinement
*wR*(*F*^2^) = 0.081	*w* = 1/[σ^2^(*F*_o_^2^) + (0.025*P*)^2^ + 2.5132*P*] where *P* = (*F*_o_^2^ + 2*F*_c_^2^)/3
*S* = 1.10	(Δ/σ)_max_ = 0.002
10618 reflections	Δρ_max_ = 0.32 e Å^−^^3^
523 parameters	Δρ_min_ = −0.39 e Å^−^^3^
72 restraints	Extinction correction: SHELXL-2019/2 (Sheldrick, 2015), Fc^*^=kFc[1+0.001xFc^2^λ^3^/sin(2θ)]^-1/4^
Primary atom site location: structure-invariant direct methods	Extinction coefficient: 0.00096 (18)

### Triphenylphosphonium trichlorido(triphenylphosphane-κP)cobaltate(II) benzene disolvate Special details

**Table:**

Geometry. All esds (except the esd in the dihedral angle between two l.s. planes) are estimated using the full covariance matrix. The cell esds are taken into account individually in the estimation of esds in distances, angles and torsion angles; correlations between esds in cell parameters are only used when they are defined by crystal symmetry. An approximate (isotropic) treatment of cell esds is used for estimating esds involving l.s. planes.

### Triphenylphosphonium trichlorido(triphenylphosphane-κP)cobaltate(II) benzene disolvate Fractional atomic coordinates and isotropic or equivalent isotropic displacement parameters (Å^2^)

**Table:**

	*x*	*y*	*z*	*U*_iso_*/*U*_eq_	Occ. (<1)
Co1	0.22726 (2)	0.57761 (2)	0.64710 (2)	0.02814 (6)	
Cl1	0.06114 (4)	0.60916 (4)	0.68130 (2)	0.04288 (10)	
Cl2	0.22832 (5)	0.67126 (4)	0.58562 (2)	0.05106 (12)	
Cl3	0.39690 (3)	0.58985 (3)	0.69482 (2)	0.03465 (9)	
P1	0.22642 (3)	0.39273 (3)	0.62985 (2)	0.02457 (8)	
P2	0.76380 (3)	0.07677 (3)	0.70830 (2)	0.02696 (8)	
C11	0.35395 (13)	0.34857 (12)	0.60188 (5)	0.0281 (3)	
C12	0.45791 (15)	0.41007 (14)	0.60449 (6)	0.0385 (4)	
H12	0.460935	0.475156	0.620143	0.046*	
C13	0.55740 (16)	0.37733 (16)	0.58444 (6)	0.0461 (4)	
H13	0.628444	0.419848	0.586463	0.055*	
C14	0.55363 (16)	0.28333 (16)	0.56157 (6)	0.0449 (4)	
H14	0.622434	0.260414	0.548221	0.054*	
C15	0.44962 (16)	0.22231 (15)	0.55807 (6)	0.0443 (4)	
H15	0.446516	0.158043	0.541851	0.053*	
C16	0.35031 (15)	0.25444 (13)	0.57805 (5)	0.0371 (3)	
H16	0.279060	0.212168	0.575542	0.045*	
C21	0.23067 (13)	0.31045 (11)	0.67874 (5)	0.0285 (3)	
C22	0.31954 (16)	0.23475 (13)	0.68989 (5)	0.0383 (4)	
H22	0.381795	0.223343	0.671485	0.046*	
C23	0.31752 (19)	0.17565 (15)	0.72793 (6)	0.0504 (5)	
H23	0.378698	0.124219	0.735571	0.060*	
C24	0.2271 (2)	0.19146 (15)	0.75457 (6)	0.0522 (5)	
H24	0.225514	0.149990	0.780278	0.063*	
C25	0.13871 (18)	0.26712 (15)	0.74423 (6)	0.0452 (4)	
H25	0.076662	0.277803	0.762785	0.054*	
C26	0.14092 (15)	0.32739 (13)	0.70666 (5)	0.0346 (3)	
H26	0.081172	0.380505	0.699816	0.042*	
C31	0.09423 (13)	0.34600 (12)	0.59506 (5)	0.0275 (3)	
C32	0.04677 (14)	0.24392 (13)	0.59722 (5)	0.0342 (3)	
H32	0.080765	0.195545	0.618810	0.041*	
C33	−0.05067 (15)	0.21306 (14)	0.56761 (6)	0.0418 (4)	
H33	−0.083390	0.143696	0.569261	0.050*	
C34	−0.09977 (15)	0.28228 (16)	0.53603 (6)	0.0441 (4)	
H34	−0.165174	0.260248	0.515607	0.053*	
C35	−0.05421 (17)	0.38338 (17)	0.53402 (6)	0.0491 (4)	
H35	−0.088706	0.431255	0.512319	0.059*	
C36	0.04195 (16)	0.41587 (14)	0.56355 (6)	0.0401 (4)	
H36	0.072192	0.486193	0.562213	0.048*	
C41	0.75381 (13)	0.21631 (12)	0.69837 (5)	0.0309 (3)	
C42	0.66069 (17)	0.27264 (14)	0.71490 (6)	0.0427 (4)	
H42	0.606567	0.237584	0.732053	0.051*	
C43	0.6477 (2)	0.37994 (15)	0.70614 (7)	0.0522 (5)	
H43	0.584456	0.418992	0.717253	0.063*	
C44	0.7270 (2)	0.43013 (15)	0.68114 (7)	0.0525 (5)	
H44	0.717348	0.503671	0.674926	0.063*	
C45	0.81943 (19)	0.37509 (16)	0.66521 (7)	0.0545 (5)	
H45	0.874090	0.410698	0.648439	0.065*	
C46	0.83296 (16)	0.26732 (15)	0.67362 (6)	0.0444 (4)	
H46	0.896399	0.228790	0.662410	0.053*	
C51	0.88873 (13)	0.01638 (12)	0.68543 (5)	0.0312 (3)	
C52	0.99972 (15)	0.01000 (17)	0.71046 (6)	0.0475 (4)	
H52	1.010991	0.041581	0.738620	0.057*	
C53	1.09425 (17)	−0.0431 (2)	0.69387 (8)	0.0626 (6)	
H53	1.170887	−0.047373	0.710744	0.075*	
C54	1.07820 (17)	−0.08970 (17)	0.65325 (7)	0.0536 (5)	
H54	1.142759	−0.127767	0.642547	0.064*	
C55	0.96903 (18)	−0.08109 (19)	0.62828 (7)	0.0576 (5)	
H55	0.958887	−0.111168	0.599828	0.069*	
C56	0.87367 (17)	−0.02901 (18)	0.64420 (6)	0.0504 (5)	
H56	0.797705	−0.024242	0.626911	0.061*	
C61	0.62667 (13)	0.01349 (12)	0.68678 (5)	0.0288 (3)	
C62	0.59340 (16)	−0.07992 (13)	0.70637 (6)	0.0399 (4)	
H62	0.637722	−0.104546	0.732526	0.048*	
C63	0.49483 (18)	−0.13659 (15)	0.68725 (7)	0.0506 (5)	
H63	0.471918	−0.200959	0.700183	0.061*	
C64	0.42989 (17)	−0.10025 (16)	0.64966 (7)	0.0503 (5)	
H64	0.363017	−0.140150	0.636573	0.060*	
C65	0.46119 (17)	−0.00624 (16)	0.63080 (7)	0.0498 (5)	
H65	0.414853	0.019029	0.605165	0.060*	
C66	0.56006 (15)	0.05141 (14)	0.64918 (6)	0.0396 (4)	
H66	0.582040	0.116057	0.636248	0.048*	
C71	0.16723 (18)	0.09182 (16)	0.49511 (7)	0.0510 (5)	
H71	0.111949	0.149263	0.490444	0.061*	
C72	0.26135 (19)	0.08187 (16)	0.46958 (6)	0.0491 (4)	
H72	0.271003	0.132172	0.447224	0.059*	
C73	0.34138 (19)	−0.00149 (18)	0.47669 (7)	0.0541 (5)	
H73	0.406099	−0.008970	0.459060	0.065*	
C74	0.3277 (2)	−0.07396 (16)	0.50928 (7)	0.0558 (5)	
H74	0.383551	−0.130888	0.514377	0.067*	
C75	0.2333 (2)	−0.06383 (16)	0.53441 (7)	0.0538 (5)	
H75	0.223327	−0.114080	0.556762	0.065*	
C76	0.15334 (19)	0.01882 (17)	0.52723 (7)	0.0536 (5)	
H76	0.087978	0.025567	0.544609	0.064*	
H2	0.7786 (16)	0.0606 (15)	0.7493 (6)	0.039 (5)*	
C81	0.5761 (3)	0.6801 (4)	0.5880 (2)	0.0570 (14)	0.580 (11)
H81	0.497748	0.694478	0.596320	0.068*	0.580 (11)
C82	0.5886 (6)	0.6418 (5)	0.54632 (17)	0.072 (2)	0.580 (11)
H82	0.518796	0.630012	0.526234	0.087*	0.580 (11)
C83	0.7031 (9)	0.6209 (5)	0.53407 (14)	0.089 (3)	0.580 (11)
H83	0.711691	0.594696	0.505620	0.107*	0.580 (11)
C84	0.8052 (5)	0.6382 (5)	0.5635 (3)	0.086 (3)	0.580 (11)
H84	0.883541	0.623847	0.555093	0.103*	0.580 (11)
C85	0.7927 (4)	0.6765 (5)	0.6051 (2)	0.070 (2)	0.580 (11)
H85	0.862496	0.688314	0.625179	0.083*	0.580 (11)
C86	0.6781 (6)	0.6975 (4)	0.61734 (9)	0.0578 (15)	0.580 (11)
H86	0.669601	0.723631	0.645793	0.069*	0.580 (11)
C91	0.7755 (7)	0.6260 (5)	0.54132 (18)	0.069 (2)	0.420 (11)
H91	0.825576	0.606100	0.519494	0.083*	0.420 (11)
C92	0.6502 (8)	0.6250 (6)	0.53201 (19)	0.066 (2)	0.420 (11)
H92	0.614659	0.604506	0.503822	0.079*	0.420 (11)
C93	0.5769 (3)	0.6541 (6)	0.5639 (4)	0.064 (2)	0.420 (11)
H93	0.491308	0.653417	0.557584	0.077*	0.420 (11)
C94	0.6290 (9)	0.6841 (5)	0.6052 (3)	0.063 (2)	0.420 (11)
H94	0.578872	0.703924	0.627016	0.075*	0.420 (11)
C95	0.7542 (10)	0.6850 (7)	0.61450 (14)	0.059 (2)	0.420 (11)
H95	0.789788	0.705519	0.642688	0.071*	0.420 (11)
C96	0.8275 (4)	0.6560 (7)	0.5826 (3)	0.065 (2)	0.420 (11)
H96	0.913141	0.656607	0.588927	0.078*	0.420 (11)

### Triphenylphosphonium trichlorido(triphenylphosphane-κP)cobaltate(II) benzene disolvate Atomic displacement parameters (Å^2^)

**Table:**

	*U*^11^	*U*^22^	*U*^33^	*U*^12^	*U*^13^	*U*^23^
Co1	0.03459 (11)	0.02290 (10)	0.02626 (10)	−0.00063 (8)	0.00009 (8)	0.00095 (7)
Cl1	0.0398 (2)	0.0481 (2)	0.0410 (2)	0.01255 (18)	0.00541 (16)	0.00370 (17)
Cl2	0.0727 (3)	0.0409 (2)	0.0380 (2)	−0.0081 (2)	−0.0017 (2)	0.01524 (18)
Cl3	0.03560 (19)	0.03528 (19)	0.03212 (18)	−0.00622 (15)	−0.00087 (14)	−0.00509 (14)
P1	0.02735 (17)	0.02210 (17)	0.02383 (16)	−0.00099 (13)	0.00065 (13)	−0.00026 (13)
P2	0.02619 (17)	0.02732 (18)	0.02725 (17)	−0.00001 (14)	0.00222 (13)	−0.00032 (14)
C11	0.0299 (7)	0.0282 (7)	0.0258 (6)	0.0007 (6)	0.0010 (5)	−0.0002 (5)
C12	0.0352 (8)	0.0399 (9)	0.0408 (9)	−0.0066 (7)	0.0064 (7)	−0.0104 (7)
C13	0.0332 (8)	0.0571 (11)	0.0489 (10)	−0.0078 (8)	0.0088 (7)	−0.0083 (9)
C14	0.0354 (9)	0.0586 (11)	0.0415 (9)	0.0084 (8)	0.0080 (7)	−0.0070 (8)
C15	0.0435 (9)	0.0434 (10)	0.0462 (9)	0.0053 (8)	0.0058 (7)	−0.0142 (8)
C16	0.0353 (8)	0.0339 (8)	0.0424 (9)	−0.0015 (6)	0.0051 (6)	−0.0089 (7)
C21	0.0329 (7)	0.0237 (7)	0.0278 (7)	−0.0033 (6)	−0.0021 (5)	0.0007 (5)
C22	0.0427 (9)	0.0328 (8)	0.0381 (8)	0.0045 (7)	−0.0018 (7)	0.0041 (7)
C23	0.0636 (12)	0.0379 (9)	0.0467 (10)	0.0067 (9)	−0.0079 (9)	0.0132 (8)
C24	0.0776 (14)	0.0404 (10)	0.0370 (9)	−0.0094 (9)	−0.0018 (9)	0.0146 (8)
C25	0.0592 (11)	0.0418 (9)	0.0357 (8)	−0.0111 (8)	0.0103 (8)	0.0054 (7)
C26	0.0391 (8)	0.0331 (8)	0.0317 (7)	−0.0036 (6)	0.0041 (6)	0.0029 (6)
C31	0.0270 (7)	0.0287 (7)	0.0268 (6)	−0.0005 (5)	0.0024 (5)	−0.0028 (5)
C32	0.0329 (8)	0.0306 (8)	0.0386 (8)	−0.0034 (6)	0.0024 (6)	−0.0009 (6)
C33	0.0351 (8)	0.0394 (9)	0.0512 (10)	−0.0115 (7)	0.0063 (7)	−0.0112 (8)
C34	0.0327 (8)	0.0573 (11)	0.0408 (9)	−0.0052 (8)	−0.0035 (7)	−0.0123 (8)
C35	0.0474 (10)	0.0544 (11)	0.0411 (9)	−0.0031 (9)	−0.0150 (8)	0.0031 (8)
C36	0.0439 (9)	0.0367 (9)	0.0372 (8)	−0.0047 (7)	−0.0073 (7)	0.0029 (7)
C41	0.0314 (7)	0.0279 (7)	0.0326 (7)	−0.0031 (6)	0.0000 (6)	−0.0030 (6)
C42	0.0504 (10)	0.0337 (9)	0.0458 (9)	0.0054 (7)	0.0131 (8)	0.0016 (7)
C43	0.0673 (13)	0.0345 (9)	0.0549 (11)	0.0120 (9)	0.0071 (9)	−0.0034 (8)
C44	0.0692 (13)	0.0286 (9)	0.0561 (11)	−0.0058 (8)	−0.0101 (10)	0.0024 (8)
C45	0.0510 (11)	0.0416 (10)	0.0704 (13)	−0.0138 (9)	0.0043 (10)	0.0144 (9)
C46	0.0365 (9)	0.0395 (9)	0.0584 (11)	−0.0044 (7)	0.0099 (8)	0.0055 (8)
C51	0.0288 (7)	0.0296 (7)	0.0354 (7)	−0.0002 (6)	0.0050 (6)	−0.0011 (6)
C52	0.0315 (8)	0.0625 (12)	0.0471 (10)	0.0037 (8)	−0.0017 (7)	−0.0157 (9)
C53	0.0301 (9)	0.0861 (16)	0.0702 (14)	0.0127 (10)	−0.0019 (9)	−0.0185 (12)
C54	0.0376 (9)	0.0579 (12)	0.0675 (13)	0.0073 (8)	0.0162 (9)	−0.0138 (10)
C55	0.0453 (10)	0.0765 (15)	0.0522 (11)	0.0044 (10)	0.0109 (9)	−0.0261 (10)
C56	0.0356 (9)	0.0727 (14)	0.0421 (9)	0.0067 (9)	0.0002 (7)	−0.0173 (9)
C61	0.0266 (7)	0.0264 (7)	0.0338 (7)	−0.0006 (5)	0.0051 (5)	−0.0018 (6)
C62	0.0462 (9)	0.0316 (8)	0.0425 (9)	−0.0049 (7)	0.0077 (7)	0.0034 (7)
C63	0.0550 (11)	0.0373 (9)	0.0620 (12)	−0.0177 (8)	0.0181 (9)	−0.0028 (8)
C64	0.0341 (9)	0.0489 (11)	0.0683 (13)	−0.0119 (8)	0.0064 (8)	−0.0172 (9)
C65	0.0380 (9)	0.0495 (11)	0.0585 (11)	−0.0020 (8)	−0.0111 (8)	−0.0044 (9)
C66	0.0373 (8)	0.0341 (8)	0.0455 (9)	−0.0032 (7)	−0.0042 (7)	0.0036 (7)
C71	0.0479 (10)	0.0447 (10)	0.0590 (12)	0.0066 (8)	−0.0009 (9)	−0.0040 (9)
C72	0.0572 (11)	0.0463 (10)	0.0427 (10)	−0.0067 (9)	0.0005 (8)	−0.0003 (8)
C73	0.0484 (11)	0.0621 (13)	0.0521 (11)	0.0019 (9)	0.0071 (9)	−0.0141 (10)
C74	0.0649 (13)	0.0432 (11)	0.0562 (12)	0.0133 (9)	−0.0086 (10)	−0.0129 (9)
C75	0.0782 (14)	0.0387 (10)	0.0430 (10)	−0.0110 (10)	−0.0008 (9)	−0.0031 (8)
C76	0.0526 (11)	0.0537 (12)	0.0561 (11)	−0.0066 (9)	0.0136 (9)	−0.0083 (9)
C81	0.060 (3)	0.057 (3)	0.053 (3)	−0.006 (2)	−0.003 (2)	0.011 (3)
C82	0.109 (5)	0.061 (3)	0.046 (3)	−0.006 (3)	0.001 (3)	0.005 (3)
C83	0.134 (8)	0.066 (4)	0.072 (4)	0.017 (6)	0.035 (4)	0.007 (3)
C84	0.099 (4)	0.059 (4)	0.107 (7)	0.028 (4)	0.040 (4)	0.017 (6)
C85	0.055 (3)	0.064 (3)	0.089 (5)	0.019 (3)	0.004 (3)	0.027 (4)
C86	0.058 (4)	0.062 (3)	0.052 (2)	0.005 (3)	−0.004 (2)	0.0156 (19)
C91	0.086 (5)	0.065 (5)	0.057 (4)	0.018 (5)	0.015 (4)	0.007 (4)
C92	0.085 (6)	0.058 (4)	0.055 (4)	0.009 (4)	0.009 (4)	0.013 (3)
C93	0.064 (4)	0.059 (5)	0.069 (7)	−0.004 (3)	0.005 (3)	0.012 (5)
C94	0.071 (5)	0.059 (4)	0.061 (5)	0.000 (5)	0.024 (4)	0.011 (4)
C95	0.072 (6)	0.060 (4)	0.047 (3)	0.001 (5)	0.016 (4)	0.011 (3)
C96	0.068 (4)	0.072 (6)	0.058 (5)	0.026 (3)	0.014 (3)	0.014 (4)

### Triphenylphosphonium trichlorido(triphenylphosphane-κP)cobaltate(II) benzene disolvate Geometric parameters (Å, º)

**Table:**

Co1—Cl2	2.2313 (5)	C52—C53	1.387 (3)
Co1—Cl1	2.2541 (5)	C52—H52	0.9500
Co1—Cl3	2.2671 (4)	C53—C54	1.376 (3)
Co1—P1	2.3893 (4)	C53—H53	0.9500
P1—C31	1.8201 (15)	C54—C55	1.369 (3)
P1—C11	1.8205 (15)	C54—H54	0.9500
P1—C21	1.8239 (15)	C55—C56	1.379 (3)
P2—C61	1.7826 (15)	C55—H55	0.9500
P2—C41	1.7861 (16)	C56—H56	0.9500
P2—C51	1.7905 (15)	C61—C66	1.390 (2)
P2—H2	1.271 (18)	C61—C62	1.390 (2)
C11—C12	1.385 (2)	C62—C63	1.384 (3)
C11—C16	1.393 (2)	C62—H62	0.9500
C12—C13	1.384 (2)	C63—C64	1.375 (3)
C12—H12	0.9500	C63—H63	0.9500
C13—C14	1.376 (3)	C64—C65	1.380 (3)
C13—H13	0.9500	C64—H64	0.9500
C14—C15	1.382 (3)	C65—C66	1.386 (2)
C14—H14	0.9500	C65—H65	0.9500
C15—C16	1.379 (2)	C66—H66	0.9500
C15—H15	0.9500	C71—C76	1.371 (3)
C16—H16	0.9500	C71—C72	1.378 (3)
C21—C22	1.389 (2)	C71—H71	0.9500
C21—C26	1.400 (2)	C72—C73	1.378 (3)
C22—C23	1.390 (2)	C72—H72	0.9500
C22—H22	0.9500	C73—C74	1.377 (3)
C23—C24	1.376 (3)	C73—H73	0.9500
C23—H23	0.9500	C74—C75	1.374 (3)
C24—C25	1.381 (3)	C74—H74	0.9500
C24—H24	0.9500	C75—C76	1.371 (3)
C25—C26	1.386 (2)	C75—H75	0.9500
C25—H25	0.9500	C76—H76	0.9500
C26—H26	0.9500	C81—C82	1.3900
C31—C36	1.390 (2)	C81—C86	1.3900
C31—C32	1.394 (2)	C81—H81	0.9500
C32—C33	1.394 (2)	C82—C83	1.3900
C32—H32	0.9500	C82—H82	0.9500
C33—C34	1.375 (3)	C83—C84	1.3900
C33—H33	0.9500	C83—H83	0.9500
C34—C35	1.374 (3)	C84—C85	1.3900
C34—H34	0.9500	C84—H84	0.9500
C35—C36	1.388 (2)	C85—C86	1.3900
C35—H35	0.9500	C85—H85	0.9500
C36—H36	0.9500	C86—H86	0.9500
C41—C46	1.381 (2)	C91—C92	1.3900
C41—C42	1.394 (2)	C91—C96	1.3900
C42—C43	1.383 (3)	C91—H91	0.9500
C42—H42	0.9500	C92—C93	1.3900
C43—C44	1.383 (3)	C92—H92	0.9500
C43—H43	0.9500	C93—C94	1.3900
C44—C45	1.372 (3)	C93—H93	0.9500
C44—H44	0.9500	C94—C95	1.3900
C45—C46	1.388 (3)	C94—H94	0.9500
C45—H45	0.9500	C95—C96	1.3900
C46—H46	0.9500	C95—H95	0.9500
C51—C52	1.384 (2)	C96—H96	0.9500
C51—C56	1.386 (2)		
			
Cl2—Co1—Cl1	112.41 (2)	C52—C51—C56	119.95 (15)
Cl2—Co1—Cl3	115.758 (19)	C52—C51—P2	119.13 (12)
Cl1—Co1—Cl3	110.480 (18)	C56—C51—P2	120.82 (12)
Cl2—Co1—P1	109.115 (18)	C51—C52—C53	119.13 (17)
Cl1—Co1—P1	106.962 (17)	C51—C52—H52	120.4
Cl3—Co1—P1	101.167 (15)	C53—C52—H52	120.4
C31—P1—C11	104.00 (6)	C54—C53—C52	120.70 (18)
C31—P1—C21	104.48 (7)	C54—C53—H53	119.7
C11—P1—C21	105.26 (7)	C52—C53—H53	119.7
C31—P1—Co1	115.47 (5)	C55—C54—C53	119.86 (17)
C11—P1—Co1	114.73 (5)	C55—C54—H54	120.1
C21—P1—Co1	111.82 (5)	C53—C54—H54	120.1
C61—P2—C41	109.98 (7)	C54—C55—C56	120.38 (18)
C61—P2—C51	109.30 (7)	C54—C55—H55	119.8
C41—P2—C51	112.81 (7)	C56—C55—H55	119.8
C61—P2—H2	108.2 (8)	C55—C56—C51	119.95 (17)
C41—P2—H2	109.1 (8)	C55—C56—H56	120.0
C51—P2—H2	107.3 (8)	C51—C56—H56	120.0
C12—C11—C16	118.95 (14)	C66—C61—C62	120.64 (15)
C12—C11—P1	119.12 (12)	C66—C61—P2	120.88 (12)
C16—C11—P1	121.93 (12)	C62—C61—P2	118.25 (12)
C13—C12—C11	120.45 (16)	C63—C62—C61	119.14 (17)
C13—C12—H12	119.8	C63—C62—H62	120.4
C11—C12—H12	119.8	C61—C62—H62	120.4
C14—C13—C12	120.15 (17)	C64—C63—C62	120.38 (17)
C14—C13—H13	119.9	C64—C63—H63	119.8
C12—C13—H13	119.9	C62—C63—H63	119.8
C13—C14—C15	119.90 (16)	C63—C64—C65	120.46 (17)
C13—C14—H14	120.0	C63—C64—H64	119.8
C15—C14—H14	120.0	C65—C64—H64	119.8
C16—C15—C14	120.18 (16)	C64—C65—C66	120.16 (18)
C16—C15—H15	119.9	C64—C65—H65	119.9
C14—C15—H15	119.9	C66—C65—H65	119.9
C15—C16—C11	120.34 (16)	C65—C66—C61	119.20 (16)
C15—C16—H16	119.8	C65—C66—H66	120.4
C11—C16—H16	119.8	C61—C66—H66	120.4
C22—C21—C26	119.10 (14)	C76—C71—C72	120.02 (19)
C22—C21—P1	123.34 (12)	C76—C71—H71	120.0
C26—C21—P1	117.53 (11)	C72—C71—H71	120.0
C21—C22—C23	120.10 (17)	C73—C72—C71	119.59 (19)
C21—C22—H22	120.0	C73—C72—H72	120.2
C23—C22—H22	120.0	C71—C72—H72	120.2
C24—C23—C22	120.16 (18)	C74—C73—C72	120.1 (2)
C24—C23—H23	119.9	C74—C73—H73	119.9
C22—C23—H23	119.9	C72—C73—H73	119.9
C23—C24—C25	120.54 (16)	C75—C74—C73	119.90 (19)
C23—C24—H24	119.7	C75—C74—H74	120.1
C25—C24—H24	119.7	C73—C74—H74	120.1
C24—C25—C26	119.70 (18)	C76—C75—C74	120.0 (2)
C24—C25—H25	120.2	C76—C75—H75	120.0
C26—C25—H25	120.2	C74—C75—H75	120.0
C25—C26—C21	120.37 (16)	C75—C76—C71	120.4 (2)
C25—C26—H26	119.8	C75—C76—H76	119.8
C21—C26—H26	119.8	C71—C76—H76	119.8
C36—C31—C32	119.06 (14)	C82—C81—C86	120.0
C36—C31—P1	117.14 (12)	C82—C81—H81	120.0
C32—C31—P1	123.75 (12)	C86—C81—H81	120.0
C33—C32—C31	119.82 (15)	C81—C82—C83	120.0
C33—C32—H32	120.1	C81—C82—H82	120.0
C31—C32—H32	120.1	C83—C82—H82	120.0
C34—C33—C32	120.47 (16)	C82—C83—C84	120.0
C34—C33—H33	119.8	C82—C83—H83	120.0
C32—C33—H33	119.8	C84—C83—H83	120.0
C35—C34—C33	119.98 (16)	C83—C84—C85	120.0
C35—C34—H34	120.0	C83—C84—H84	120.0
C33—C34—H34	120.0	C85—C84—H84	120.0
C34—C35—C36	120.33 (17)	C86—C85—C84	120.0
C34—C35—H35	119.8	C86—C85—H85	120.0
C36—C35—H35	119.8	C84—C85—H85	120.0
C35—C36—C31	120.32 (16)	C85—C86—C81	120.0
C35—C36—H36	119.8	C85—C86—H86	120.0
C31—C36—H36	119.8	C81—C86—H86	120.0
C46—C41—C42	120.28 (16)	C92—C91—C96	120.0
C46—C41—P2	121.40 (13)	C92—C91—H91	120.0
C42—C41—P2	118.28 (12)	C96—C91—H91	120.0
C43—C42—C41	119.53 (18)	C93—C92—C91	120.0
C43—C42—H42	120.2	C93—C92—H92	120.0
C41—C42—H42	120.2	C91—C92—H92	120.0
C44—C43—C42	119.73 (19)	C92—C93—C94	120.0
C44—C43—H43	120.1	C92—C93—H93	120.0
C42—C43—H43	120.1	C94—C93—H93	120.0
C45—C44—C43	120.85 (18)	C95—C94—C93	120.0
C45—C44—H44	119.6	C95—C94—H94	120.0
C43—C44—H44	119.6	C93—C94—H94	120.0
C44—C45—C46	119.88 (19)	C94—C95—C96	120.0
C44—C45—H45	120.1	C94—C95—H95	120.0
C46—C45—H45	120.1	C96—C95—H95	120.0
C41—C46—C45	119.72 (18)	C95—C96—C91	120.0
C41—C46—H46	120.1	C95—C96—H96	120.0
C45—C46—H46	120.1	C91—C96—H96	120.0
			
C31—P1—C11—C12	147.12 (13)	C41—C42—C43—C44	0.0 (3)
C21—P1—C11—C12	−103.30 (13)	C42—C43—C44—C45	−0.6 (3)
Co1—P1—C11—C12	20.06 (14)	C43—C44—C45—C46	0.9 (3)
C31—P1—C11—C16	−33.13 (14)	C42—C41—C46—C45	−0.1 (3)
C21—P1—C11—C16	76.45 (14)	P2—C41—C46—C45	177.51 (15)
Co1—P1—C11—C16	−160.20 (12)	C44—C45—C46—C41	−0.6 (3)
C16—C11—C12—C13	−1.3 (3)	C61—P2—C51—C52	−147.38 (15)
P1—C11—C12—C13	178.47 (14)	C41—P2—C51—C52	89.93 (16)
C11—C12—C13—C14	0.2 (3)	C61—P2—C51—C56	28.95 (17)
C12—C13—C14—C15	1.0 (3)	C41—P2—C51—C56	−93.74 (16)
C13—C14—C15—C16	−1.1 (3)	C56—C51—C52—C53	−0.7 (3)
C14—C15—C16—C11	0.0 (3)	P2—C51—C52—C53	175.67 (18)
C12—C11—C16—C15	1.2 (2)	C51—C52—C53—C54	−0.5 (4)
P1—C11—C16—C15	−178.58 (14)	C52—C53—C54—C55	1.9 (4)
C31—P1—C21—C22	111.23 (14)	C53—C54—C55—C56	−2.2 (4)
C11—P1—C21—C22	2.00 (15)	C54—C55—C56—C51	1.0 (4)
Co1—P1—C21—C22	−123.19 (13)	C52—C51—C56—C55	0.4 (3)
C31—P1—C21—C26	−70.46 (13)	P2—C51—C56—C55	−175.85 (17)
C11—P1—C21—C26	−179.69 (12)	C41—P2—C61—C66	33.31 (15)
Co1—P1—C21—C26	55.12 (12)	C51—P2—C61—C66	−91.06 (14)
C26—C21—C22—C23	1.1 (2)	C41—P2—C61—C62	−152.07 (13)
P1—C21—C22—C23	179.39 (14)	C51—P2—C61—C62	83.57 (14)
C21—C22—C23—C24	0.4 (3)	C66—C61—C62—C63	1.8 (3)
C22—C23—C24—C25	−1.0 (3)	P2—C61—C62—C63	−172.82 (14)
C23—C24—C25—C26	0.2 (3)	C61—C62—C63—C64	−0.7 (3)
C24—C25—C26—C21	1.3 (3)	C62—C63—C64—C65	−0.8 (3)
C22—C21—C26—C25	−2.0 (2)	C63—C64—C65—C66	1.3 (3)
P1—C21—C26—C25	179.66 (13)	C64—C65—C66—C61	−0.2 (3)
C11—P1—C31—C36	−92.74 (13)	C62—C61—C66—C65	−1.4 (3)
C21—P1—C31—C36	157.11 (13)	P2—C61—C66—C65	173.14 (14)
Co1—P1—C31—C36	33.86 (14)	C76—C71—C72—C73	−0.2 (3)
C11—P1—C31—C32	84.52 (14)	C71—C72—C73—C74	−0.4 (3)
C21—P1—C31—C32	−25.63 (14)	C72—C73—C74—C75	0.8 (3)
Co1—P1—C31—C32	−148.88 (11)	C73—C74—C75—C76	−0.5 (3)
C36—C31—C32—C33	0.8 (2)	C74—C75—C76—C71	−0.2 (3)
P1—C31—C32—C33	−176.37 (12)	C72—C71—C76—C75	0.5 (3)
C31—C32—C33—C34	0.5 (3)	C86—C81—C82—C83	0.0
C32—C33—C34—C35	−1.2 (3)	C81—C82—C83—C84	0.0
C33—C34—C35—C36	0.5 (3)	C82—C83—C84—C85	0.0
C34—C35—C36—C31	0.9 (3)	C83—C84—C85—C86	0.0
C32—C31—C36—C35	−1.6 (3)	C84—C85—C86—C81	0.0
P1—C31—C36—C35	175.84 (15)	C82—C81—C86—C85	0.0
C61—P2—C41—C46	−119.37 (14)	C96—C91—C92—C93	0.0
C51—P2—C41—C46	2.93 (16)	C91—C92—C93—C94	0.0
C61—P2—C41—C42	58.23 (15)	C92—C93—C94—C95	0.0
C51—P2—C41—C42	−179.46 (13)	C93—C94—C95—C96	0.0
C46—C41—C42—C43	0.4 (3)	C94—C95—C96—C91	0.0
P2—C41—C42—C43	−177.28 (15)	C92—C91—C96—C95	0.0

### Triphenylphosphonium trichlorido(triphenylphosphane-κP)cobaltate(II) benzene disolvate Hydrogen-bond geometry (Å, º)

*Cg*1–*Cg*4 are the centroids of the (C21–C26), (C81–C86), (C91–C96) ring and (C11–C16) rings, respectively.

**Table:**

*D*—H···*A*	*D*—H	H···*A*	*D*···*A*	*D*—H···*A*
P2—H2···Cl1^i^	1.271 (18)	2.701 (18)	3.7531 (6)	138.9 (11)
P2—H2···Cl3^i^	1.271 (18)	2.758 (18)	3.6380 (6)	124.9 (11)
C52—H52···Cl1^i^	0.95	2.80	3.6874 (19)	155
C63—H63···Cl3^ii^	0.95	2.76	3.6294 (19)	152
C75—H75···Cl2^ii^	0.95	2.85	3.694 (2)	149
C85a—H85a···Cl1^iii^	0.95	2.83	3.690 (3)	150
C62—H62···*Cg*1^i^	0.95	2.72	3.6042 (19)	154
C72—H72···*Cg*2^iv^	0.95	2.77	3.611 (3)	148
C72—H72···*Cg*3^iv^	0.95	2.78	3.607 (4)	146
C92—H92···*Cg*4^iv^	0.95	2.84	3.636 (6)	143

Symmetry codes: (i) −*x*+1, *y*−1/2, −*z*+3/2; (ii) *x*, *y*−1, *z*; (iii) *x*+1, *y*, *z*; (iv) −*x*+1, −*y*+1, −*z*+1.
